# Autophagy in Heart Failure: Insights into Mechanisms and Therapeutic Implications

**DOI:** 10.3390/jcdd10080352

**Published:** 2023-08-18

**Authors:** Magdalena Bielawska, Marta Warszyńska, Monika Stefańska, Przemysław Błyszczuk

**Affiliations:** 1 Department of Clinical Immunology, Jagiellonian University Medical College, University Children’s Hospital, Wielicka 265, 30-663 Cracow, Poland; magdalena.bielawska@doctoral.uj.edu.pl (M.B.);; 2 Department of Rheumatology, University Hospital Zurich, University of Zurich, 8952 Schlieren, Switzerland

**Keywords:** autophagy, heart failure, molecular mechanisms, pharmacological interventions

## Abstract

Autophagy, a dynamic and complex process responsible for the clearance of damaged cellular components, plays a crucial role in maintaining myocardial homeostasis. In the context of heart failure, autophagy has been recognized as a response mechanism aimed at counteracting pathogenic processes and promoting cellular health. Its relevance has been underscored not only in various animal models, but also in the human heart. Extensive research efforts have been dedicated to understanding the significance of autophagy and unravelling its complex molecular mechanisms. This review aims to consolidate the current knowledge of the involvement of autophagy during the progression of heart failure. Specifically, we provide a comprehensive overview of published data on the impact of autophagy deregulation achieved by genetic modifications or by pharmacological interventions in ischemic and non-ischemic models of heart failure. Furthermore, we delve into the intricate molecular mechanisms through which autophagy regulates crucial cellular processes within the three predominant cell populations of the heart: cardiomyocytes, cardiac fibroblasts, and endothelial cells. Finally, we emphasize the need for future research to unravel the therapeutic potential associated with targeting autophagy in the management of heart failure.

## 1. Introduction

Heart failure is a condition in which the heart has difficulty pumping enough blood to meet the body’s needs. It is a progressive disease that, if left untreated, often leads to hemodynamic collapse and death. While there have been significant advances in the treatment of heart failure in recent years, there are still no successful therapies. These restrictions include medication side effects, comorbidities that reduce the effectiveness of medications, and a lack of effective treatment options for advanced heart failure patients. There is therefore an important, unmet clinical need for developing novel therapeutic strategies to prevent or slow down the progression of heart disease. Autophagy is a cellular process in which cells degrade and recycle their own components. The dysregulation of autophagy has been implicated in the development of many cardiovascular diseases. As a result, there has been growing interest in targeting autophagy as a therapeutic approach for cardiac disease. In this review, we describe the current knowledge regarding the contribution of autophagy to the development of heart failure and assess the therapeutic potential of pharmacological autophagy modulators.

## 2. Heart Failure

Heart failure is characterized as a clinical syndrome caused by a structural and/or functional cardiac abnormality, leading to reduced cardiac output and/or elevated intracardiac pressures at rest or during stress [1]. This disease affects 26 million people worldwide and represents a leading cause of death globally [2]. The Western diet, lifestyle, and population aging are to blame for the steadily increasing incidence of heart disease, which accounts for 1 million hospitalizations in the United States and Europe each year [2,3]. Heart failure can start quickly or develop slowly over time, depending on the cause. Sudden onset can occur following an acute ischemic episode in the cardiac muscle, called myocardial infarction. In this scenario, restricted oxygen supply causes the death of cardiomyocytes (CMs), which are replaced by mechanically dysfunctional fibrotic scars. In a non-ischemic condition, asymptomatic changes in the cardiac muscle can advance to a dilated or hypertrophic cardiomyopathy phenotype, in which the ventricles become enlarged and stiff. In cardiomyopathies, cardiac insufficiency is primarily observed in the left ventricle. Right ventricular failure is less frequent and is commonly associated with pulmonary hypertension or chronic lung disease.

Changes in the architecture of the failing heart are frequently associated with abnormal heart tissue remodelling. Fibrotic lesions, an increase in the size and form of CMs, and, occasionally, the presence of inflammatory cells are typical morphological alterations observed in the myocardium of heart failure patients.

Cardiac fibrosis refers to the excessive accumulation of stromal cells and extracellular matrix (ECM) components in the myocardium. In a case of myocardial infarction, a fibrotic scar initially supports the integrity of the damaged tissue and regenerative processes, but in the long term, it contributes to organ failure. In non-ischemic heart disease, pathological tissue remodeling and fibrogenesis, on the other hand, show no benefit and are recognized as pathogenic processes that promote disease progression. Mechanistically, fibrosis causes a stiffening of the cardiac tissue and thereby impairs cardiac performance, leading to systolic and diastolic dysfunctions. Furthermore, cardiac fibrosis interferes with the physiological propagation of electrical impulses, increasing the risk of serious arrhythmic events [4]. Resident cardiac fibroblasts represent key cellular players in profibrotic processes in the heart. In addition to fibroblasts, other cell types also contribute to the progression of cardiac fibrosis. Endothelial cells (ECs) undergoing endothelial-to-mesenchymal transition represent an example of an alternative cellular source of fibroblastic cells in the injured tissue [5]. Inflammatory cells have also been suggested to play an important role in pathological tissue remodeling in the heart. Circulating fibrocytes, ECs, and inflammatory myeloid cells have been shown to acquire a fibroblast-like and myofibroblast-like phenotype and to produce ECM during cardiac fibrogenesis [6,7,8].

The stiffening of fibrotic tissue, combined with CMs loss, results in compromised heart muscle contractility. The growth of surviving CMs (hypertrophy) represents a compensatory mechanism aiming to maintain the pumping potential of the failing heart. In the long run, this compensatory mechanism is, however, insufficient, and CMs from heart failure patients eventually show decreased contractility force.

Inflammation represents another process associated with the development and progression of heart failure [9]. Cardiac inflammation is characterized by the accumulation and activation of immune cells, as well as the release of cytokines and chemokines in the heart tissue. It occurs in post-ischemic myocardial events, but also can be caused by a variety of factors, including cardiotropic infections, toxic substances, certain medical conditions, or physical stress. Inflammatory processes in the heart can cause direct injury to CMs, promote fibrogenesis, and lead to an increase in oxidative stress. The presence of persistent inflammation in heart failure patients has also been associated with a worse prognosis [10].

Furthermore, pathological processes ongoing in the cardiac tissue negatively affect ECs and can cause endothelial dysfunction [11]. As a result, blood vessels can become constricted and coronary microcirculation may be reduced, leading to decreased cardiac output and an increased risk of ischemic events. In fact, patients with heart failure (of both ischemic and non-ischemic origin) show impaired coronary blood flow [12].

## 3. Autophagy—Overview

Cellular homeostasis requires a perfect balance between synthesis and degradation. A process of autophagy (“self-eating”), also called autophagocytosis, represents a major degradative process in eucaryotic organisms. In this process, cytosolic materials (organelles, long-lived or misfolded proteins, protein aggregates, etc.) are delivered to the endo-lysosomal system for subsequent degradation. Depending on the type of inducing signal, type of cargo, the mechanism of sequestration, and their selectivity, autophagy can be divided into three subclasses: macroautophagy, microautophagy, and chaperone-mediated autophagy [13]. A process of macroautophagy is initiated by the formation of a transient double-membrane structure called a phagophore. Phagophores play an active role in the sequestering of cytoplasm and the formation of autophagosomes, a spherical structure containing cytoplasmic cargo. Further, the autophagosome fuses with the lysosome, forming the autophagosome, and its cargo is degraded by proteases, glycosidases, sulfatases, and other lysosomal enzymes. This type of autophagy provides nutrients for the cell and controls essential housekeeping functions, such as the elimination of excessive and damaged organelles, protein aggregates, and invading microorganisms [14]. In the case of microautophagy, cytoplasmic contents enter the lysosome through an invagination of the lysosomal membrane [15]. The third type of autophagy refers to chaperone-mediated autophagy, which is a highly selective process controlled by lysosomal-associated membrane protein 2A (Lamp-2a) and chaperon complexes [16]. In this review, we will focus on macroautophagy and refer to this process as autophagy.

## 4. Molecular Mechanisms of Autophagy

Autophagy is a multistep process, tightly controlled by proteins of the autophagy-related (Atg) family. The first step in autophagosome biogenesis is the activation of the Ulk1 kinase complex and the formation of a cup-shaped double membrane structure called the phagophore or isolation membrane [17]. The scaffold protein family kinase-interacting proteins of 200 kDa (Fip200), Ulk1 and Ulk2 kinases, Atg13, and Atg101 represent the main components of the Ulk1 kinase complex. In response to pro-autophagic stimuli, Ulk1/2 kinases phosphorylate Atg13 and Fip200 and recruit the Ulk1 complex as well as the class III phosphatidylinositol 3-kinase complex to the phagophore assembly site (PAS). The sources of PAS are vesicles derived from the Golgi apparatus, which are transported by the transmembrane protein Atg9 [18]. The recruited class III phosphatidylinositol 3-kinase complex comprises the vacuolar protein sorting (Vps) kinases Vps34 and Vps15, Beclin-1 and other proteins, and is involved in the production of phosphatidylinositol-3-phosphate (PI3P) at the PAS [19]. In particular, Vps34, class III phosphoinositide 3-kinase, is the enzyme converting phosphatidylinositol to PI3P [20]. PI3P is crucial for the recruitment of a number of Atg proteins to the PAS [21]. Interaction between Beclin-1 and Vps34 promotes the catalytic activity of Vps34 and increases PI3P levels [22].

The process of phagophore elongation is orchestrated by two ubiquitin-like conjugation systems: the Atg12-Atg5-Atg16 complex and members of the Atg18 family, such as microtubule-associated protein light chain 3 (Lc3) [13,18]. In autophagocytosis, pro-Lc3 is proteolytically cleaved by Atg4 protease to generate Lc3-I, which is further conjugated to phospholipid phosphatidylethanolamine by Atg7 (E1-like enzyme), Atg3 (E2-like enzyme), and Atg12-Atg5-Atg16 complex (E3-like enzyme) [23]. Such a lipidated form of Lc3-I binds to the newly formed autophagosome membranes and is referred to as Lc3-II. The recruitment and integration of the lipidated Lc3-I into the growing phagophore depend on the Atg12-Atg5-Atg16 complex [24]. Lc3-II is found on the internal and external surfaces of the autophagosome, where it plays a role in the hemifusion of membranes and in selecting cargo for degradation [24].

Phagophore elongation leads to the fusion of the two ends of the growing PAS, generating a closed autophagosome with inner and outer membranes. It is not yet clear which proteins control this process; however, Atg9, Atg2, and Atg18 are found on the edges of the PAS [25]. During the maturation process, after the closure of the autophagosome, PIP3, Lc3, and other Atg proteins remaining on its surface are removed and recycled. PI3Ps are removed from the membrane by converting them into phosphatidylinositol-3,5,-biphosphate through FYVE finger-containing phosphoinositide kinase [26,27]. In the case of Lc3, Atg4 delipidises Lc3-II incorporated into the developing autophagosome and converts it into soluble Lc3-I, and Lc3 is thereby removed from the mature autophagosome [28]. Of note, intracellular Lc3-II levels, or the Lc3-II/Lc3-I ratio, are commonly used to monitor the active autophagy process.

The formation of the autolysosome by the fusion of the mature autophagosome with the lysosome and the subsequent enzymatic lysis of the autolysosomal content represents the final step of the autophagy process. Autophagosomes and lysosomes are localized in different cellular compartments; therefore, autophagosomes are transported to perinuclearly located lysosomes. This process is mediated by microtubules, and small GTP binding proteins like Rab7 on the surface of the autophagosome enable linking autophagosomes with microtubules [29]. The actual fusion of the autophagosome with the lysosome requires the bridging of the membranes. The efficiency and specificity of this process depend on the formation of SNARE and tether complexes [30]. Autophagosomal Stx17 and Snap19 bound to lysosomal Vamp8 represent an example of a SNARE complex. Furthermore, Hops, Plekhm1, Epg5, Tecpr1, Lc3, PIP3, Rab7, and other proteins form additional complexes to tether and fuse the autophagosome with the lysosome. A successful autophagolysosome formation is followed by the disassembly and recycling of the SNARE and tether proteins. In summary, on a molecular level, autophagy is a complex and highly organized process orchestrated by multiple proteins.

## 5. Physiological Regulation of Autophagy

Autophagy is a strictly regulated process. It is typically induced by starvation, growth factor deprivation, endoplasmic reticulum stress, or pathogen infections. The mammalian target of rapamycin (mTOR), a serine/threonine protein kinase, plays a key role in switching on and off the autophagy process in a cell. Two functional complexes, mTOR complex 1 (mTORC1) and mTOR complex 2 (mTORC2), form the catalytic subunit of this highly conserved across-species kinase. Each of these complexes is implicated in regulating specific cellular processes, but both act as negative regulators of autophagy [31]. Under normal conditions, mTOR phosphorylates Ulk1 and Atg13, preventing the formation of the Ulk1 kinase complex. mTORC1 is a main sensor of nutrient levels, which, under starving conditions, becomes less active, allowing for increased Ulk1 kinase complex activity and initiating the autophagy process [32]. Adenosine monophosphate kinase (AMPK) represents another molecular energy sensor in a cell, which under low nutrient conditions becomes phosphorylated and activated by liver kinase B1 [33]. AMPK senses the ATP/AMP ratio and controls energy homeostasis by regulating cellular metabolism. Once activated, AMPK can inhibit mTORC1 activity and thereby initiate autophagocytosis [34].

Autophagy can be induced by nutrient deprivation but can also occur in nutrient-rich conditions. The absence of certain growth factors or changes in intracellular levels of ROS represent examples of such pro-autophagic triggers. The phosphatidylinositol 3-kinase (PI3K)-Akt pathway plays an essential role in these processes. PI3K-Akt represents a well-known upstream regulator of mTORC1. On the one hand, under oxidative stress, the pro-autophagic β catalytic subunit of PI3K is activated, suppressing Akt kinase and mTORC1 activity [35]. On the other hand, growth factors such as insulin-like growth factor-1 can also activate the PI3K-Akt pathway and thereby prevent the pro-autophagic response [36]. The growth factor-mediated activation of the Akt kinase has been recognized to target mainly mTORC2 [37].

The induction of autophagy is followed by a rapid transcriptional up-regulation of autophagy-associated genes. This response is controlled by a number of autophagic transcription factors, and their activity is regulated by the mTOR, AMPK, and PI3K-Akt pathways. Transcription factor EB (Tfeb), a zinc-finger protein with KRAB and SCAN domains 3 (ZKSCAN3), and transcription factors of the forkhead box O (Foxo) family have been shown to play a particularly important role in the transcriptional regulation of autophagy. In fact, the overexpression of one of the key transcription factors is sufficient to initiate autophagocytosis [38]. Mechanistically, Tfeb and Foxo act as gene activators that, following autophagy induction, translocate to the nucleus to promote the expression of autophagy-associated genes [39]. In steady state, the expression of many of those genes is additionally repressed by repressors binding to promoter regions. In the case of ZKSCAN3, pro-autophagic signaling triggers the migration of this repressor from the nucleus to the cytoplasm, enabling the transcriptional activity of Tfeb [40]. It should be noted that the transcriptional regulation of autophagy is far more complex. A thorough description of this mechanism can be found in the dedicated review [41].

## 6. Pharmacological Modulation of Autophagy

For many years, the molecular mechanisms of action of many drugs remained unknown. It has been discovered that some of those drugs, such as rapamycin, chloroquine, or metformin, interfere with key components of the autophagy machinery, and therefore they can be used to inhibit or activate autophagocytosis independently of nutrient availability. Rapamycin represents a classical example of a drug generated before the discovery of autophagy, but with a mode of action that targets a central regulator of this process. Rapamycin is an antifungal agent obtained from *S. hygroscopicus* with strong immunosuppressive properties [42]. As indicated by its name, mTOR represents a target for rapamycin. Mechanistically, rapamycin forms a complex with FK506-binding protein 12, which negatively regulates mainly mTOR1 activity [43]. The ability to suppress mTOR activity makes rapamycin a commonly used autophagy inducer.

As discussed above, the PI3K-Akt and AMPK pathways play important roles in regulating mTOR activity. 3-methyladenine (3-MA) and wortmannin can inhibit the activity of class III PI3K, and therefore they can be used as autophagy inhibitors [44,45]. Both compounds suppress autophagy under starvation; however, 3-MA can promote autophagic flux under nutrient-rich conditions [46]. On the other hand, AMPK is a target for the anti-hyperglycemic drug metformin [47]. Metformin can activate AMPK and AMPK-dependent pathways and thereby induce autophagy [48]. Similar to metformin, resveratrol, a natural polyphenolic compound, is known to enhance autophagic flux by activating through the AMPK pathway [49].

**Figure 1:**
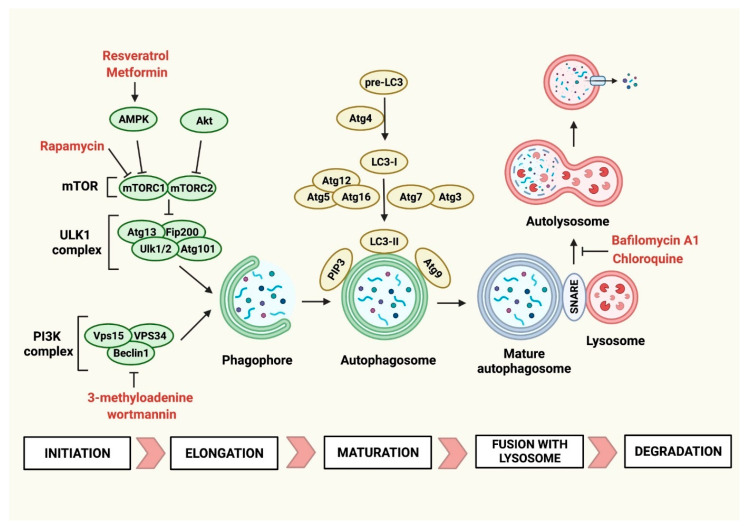
The schematic representation of the autophagy process with the indicated sites of action of selected pharmacological autophagy modulators (dark red font).

In addition to compounds targeting mTOR and mTOR upstream regulators, a process of autophagy can be modulated at the stage of autophagosome formation/maturation, its fusion with the lysosome, or the degradation of the autophagolysosomal content. The antimalarial drug chloroquine represents an example of an autophagy inhibitor impairing the fusion of the autophagosome with the lysosome [50]. Chemically, chloroquine is considered a weak base, so it is able to raise the pH level of the environment, for example, in the cellular components. Therefore, it is assumed that chloroquine blocks autophagy by interacting with lysosomes and increasing their pH, resulting in the inhibition of hydrolase activity [51]. Bafilomycin A1, like chloroquine, prevents the formation of functional autophagolysosomes. Bafilomycin A1, however, not only inhibits autophagosome-lysosome fusion but also blocks the proton pump V-ATPase responsible for low intralysosomal pH, which is essential for resident hydrolase activity [52]. The blockade of the autophagic process at the stage of the autophagolysosome results in the accumulation of a large number of misfolded proteins and damaged organelles in the cell. The schematic representation of the mode of action of the presented pharmacological autophagy regulators is illustrated in Figure 1.

In this paragraph, we listed only the most commonly used drugs modulating autophagocytosis and focused on compounds widely used in the clinic and in research, but the list of available pharmacological autophagy modulators is much longer [53]. It should, however, be noted that most of these drugs show pleiotropic effects, affecting more than one molecular target. They are often unspecific for autophagy, and therefore the effects of their action might also be mediated by off-target mechanisms. The identification of the molecular machinery controlling autophagocytosis allows for the design of novel classes of pharmacological compounds to specifically modulate this process. At the moment, such highly specific pharmacological activators or inhibitors are not commercially available.

## 7. Autophagy in Cardiac Homeostasis

As was previously mentioned, autophagy regulates a variety of cellular functions, including organelle turnover, the recycling of damaged parts, and the maintenance of cellular homeostasis. A growing body of evidence indicates that the dysregulation of autophagy may result in pathogenic changes in the structure and function of the cardiac muscle. In humans, defective autophagy caused by a mutation in the *LAMP2* gene (encoding lysosomal-associated membrane protein-2; LAMP-2) leads to Danon disease, manifested by cardiomyopathy and cardiac dysfunction as major symptoms of the disease [54]. The relevance of the proautophagic *Lamp2* in cardioprotection has been observed in mice, too. In fact, *Lamp2*^−/−^ mice show high premature mortality, and the surviving animals develop pathological changes not only in the heart, arteries, and skeletal muscle but also in non-muscular organs such as the pancreas and liver [55,56]. Cardiac pathology due to reduced autophagic activity was also found in mice with constitutive knock-outs of *Atg5*, *Gsk3a*, or *Fbxo32* (gene encoding atrogin-1). The *Atg5*^−/−^ mice showed a disorganized sarcomere structure and collapsed mitochondria in cardiac tissue, leading to the development of a cardiomyopathy phenotype and systolic dysfunction in aged animals [57]. The genetic knock-out of *Gsk3a* led on a cellular level to the accumulation of vacuole, sarcomere disarray, and swollen and disrupted mitochondria, resulting in CMs loss, cardiomyopathy associated with cardiac dysfunction, and a shortened mouse lifespan [58]. Similarly, mice lacking atrogin-1 showed an intracellular accumulation of protein aggregates and CMs apoptosis and developed age-related cardiomyopathy [59]. Another example of the cardioprotective role of autophagy was demonstrated in mice expressing the human mutation of heat shock protein family B member 6 (HSPB6). The *HSPB6^S10F^* mice showed reduced autophagy due to impaired interaction between mutated HSPB6 and Beclin-1, which in turn led to heart failure and early death [60]. Furthermore, the chronic activation of Akt in CMs effectively suppressed autophagy in these cells and caused aging-associated cardiac hypertrophy, interstitial fibrosis, and contractile dysfunction in transgenic mice [61]. In line with these data, cardiomyopathy, severe contractile dysfunction, and premature deaths have also been observed in mice with an inducible conditional knockout of *Atg5* or *Atg7* in CMs [62,63]. Furthermore, the overexpression of miR-199a in CMs suppressed autophagy, which resulted in cardiac hypertrophy [64]. Altogether, these data suggest that under homeostatic conditions, baseline autophagic regulation in CMs is needed to maintain cellular function and cardiac physiology and protect from enhanced aging. This concept has been strengthened by observations demonstrating that the overexpression of *Atg5* or the disruption of the beclin1-BCL2 autophagy regulatory complex can extend mouse lifespan [65,66]. However, it should be noted that increased autophagy in an instressed heart can also be deleterious, as shown by heart atrophy in mice with overexpressed *Foxo3* in CMs [67]. This is supported by the finding that mice overexpressing the autophagy inducer DNA-damage-inducible transcript 4-like developed impaired cardiac function [68].

Physical exercises represent an exceptional form of physiological stress that can activate a variety of signaling pathways in the heart known to regulate autophagy, such as the AMPK and mTOR pathways. The activation of these pathways can result in increased autophagy in cardiac cells, which in turn can aid in clearing damaged cellular components and promoting healthy cellular function. The expression of autophagy markers can vary depending on the type of exercise and whether it is preceded by a preparatory period. In mouse hearts, exhaustive exercise has been associated with a decrease in the levels of autophagy markers, while endurance and strength exercises have suggested activation of autophagy [69]. Studies with rats have shown that exercise preconditioning may have a cardioprotective effect by reducing the area of ischemia-hypoxia injury caused by exhaustive exercise [70]. Furthermore, it has been demonstrated that the cardioprotective effects of exercise in obese mice require intact autophagy in CMs [71].

## 8. Autophagy in Cardiac Injury

### 8.1. Myocardial Infarction

Cardiac stress or injury, of virtually any type, can activate autophagy either by nutrient deprivation or as a response to ischemia, mechanical or oxidative stress, and inflammation. In cases of cardiac ischemia, shortages in oxygen and nutrient delivery rapidly trigger autophagocytosis, particularly in surviving CMs [72,73,74]. Published data from animal models suggest that the activation of autophagy in CMs due to ischemia plays a cardioprotective role. It has been reported that the intracardiac injection of HMGB1 induced autophagy and improved CMs survival in the peri-infarcted area [75]. In line with these findings, starvation or treatment with proautophagic rapamycin or metformin reduced infarct size, while the blockage of autophagy with bafilomycin A1 resulted in the opposite effect in a model of permanent coronary artery occlusion [72,76,77]. Furthermore, the blockade of small heat shock protein 20 (Hsp20) phosphorylation suppressed autophagic activity in the infarcted heart and led to increased infarct size [78]. In line with these findings, the overexpression of Hsp20 resulted in a cardioprotective effect [79].

Reperfusion, the restoration of blood flow to the damaged myocardium following ischemic cardiac events, is a common occurrence in humans. However, this process often worsens cellular damage and exacerbates the consequences of the initial ischemic event. It has been suggested that autophagocytosis, which is activated during ischemia, is further enhanced during the reperfusion phase. Early studies conducted on explanted rabbit hearts reported an increase in autophagic vacuoles under hypoxic conditions. Subsequently, upon reoxygenation, repair processes were activated, and there was an increase in the number of lysosomes in the surviving CMs [80]. More recent data, however, point to reduced autophagy during the reperfusion phase [81]. These findings might indicate distinct molecular mechanisms controlling cardiac autophagy during ischemic events and the reperfusion phase. According to published findings, autophagy is activated during the ischemic phase through an AMPK-dependent mechanism. Conversely, in the context of ischemia-reperfusion, autophagy induction is AMPK-independent and involves Beclin 1 [82]. The overexpression of Beclin 1 in the ischemia-reperfusion model has been shown to enhance autophagic flux and significantly reduce the activation of pro-apoptotic genes [83]. However, it is worth noting that overexpression of Beclin 1 during the late phase of ischemia-reperfusion has been associated with worsened injury [84]. Instead, in the case of short ischemic periods, pharmacological autophagy activators rapamycin and metformin effectively reduced infarct size [81,85]. In line with these findings, treatment with the autophagy inhibitor wortmannin resulted in an enlarged infarct size and enhanced apoptosis [85].

In the postinfarcted heart, damaged cardiac tissue is infiltrated by inflammatory cells and, on follow-up, is replaced by a fibrotic scar, leading to impaired function of the organ. A growing body of evidence suggests that the activation of autophagy limits postinfarction inflammation, pathological tissue remodeling, and cardiac dysfunction. Autophagy inducers rapamycin, metformin, atorvastatin, or trehalose indeed showed beneficial effects by reducing fibrotic scarring and improving cardiac function in the acute myocardial infarction model [74,86,87,88,89]. In line with these findings, treatment with the pharmacological inhibitor 3-MA exacerbated postinfarction cardiac remodeling and dysfunction [74]. Likewise, *Atg7* knockdown in CMs exaggerated systolic dysfunction and cardiac fibrosis in the postinfarction heart [62]. Furthermore, *Becn1*^+/−^ mice characterized by reduced autophagic activity were reported to develop more fibrotic scars and show worse cardiac function [90], although this phenotype was not confirmed by another study [91]. These data could suggest that autophagy protects against an excessive profibrotic response and cardiac dysfunction in the postinfarcted heart. However, it should be noted that the type of these changes in the postinfarcted heart is dependent on the extent of the cellular damage caused by ischemia and reperfusion. Therefore, the exact role of autophagy in the postinfarcted heart remains elusive.

On the other hand, there are also examples of cardioprotective interventions in infarction models that were associated with suppressed cardiac autophagy. For example, the genetic downregulation of Rubicon involved in autophagy regulation resulted in reduced ischemia-reperfusion injury [84]. Similarly, the cardioprotective effect of aspirin in the permanent ligation model of myocardial infarction was associated with suppressed autophagy [92]. Additionally, treatment with long non-coding RNA, termed cardiac autophagy inhibitory factor, suppressed cardiac autophagy and attenuated myocardial infarction [93]. In line with these data, the knockdown of inhibitor of nuclear factor-κB kinases ε (IKKε) resulted in excessive autophagy in CMs and worsened cardiac function in the postinfarction heart [94].

Summarizing, most studies suggest that autophagy limits rather than exaggerates myocardial damage, postinfarction fibrosis, and heart failure. However, so far, the clinical data do not entirely support these encouraging conclusions. Treatment with proautophagic metformin showed no beneficial effects on infarct size in diabetic patients presenting ST-elevation myocardial infarction [95], although metformin was reported to reduce infarct size in diabetic rats [96]. Though metformin shows no cardioprotection in myocardial infarction, it reduces cardiovascular mortality and the incidence of heart failure in patients with cardiovascular diseases [97].

### 8.2. Pressure Overload and Hypertension

Ischemic events are important autophagy triggers in the heart. Although in non-ischemic heart disease, cardiac tissue is not challenged by episodes of oxygen or nutrient deficiency, certain pathogenic processes in the affected heart can also trigger autophagy. Increased cardiac afterload represents an example of such a trigger. Transverse aortic constriction (TAC) surgery and the continuous infusion of potent vasoconstrictor Ang II are two common methods to increase aortic pressure, against which the left ventricle pumps the blood. Both models show that increasing cardiac afterload results in hypertrophy of CMs, perivascular fibrosis, cardiomyopathy, and cardiac dysfunction. These pathological processes are associated with activated autophagocytosis in the cardiac muscle. In the TAC model, elevated levels of autophagy markers have been reported 3–4 weeks after the surgical procedure [98,99,100,101,102,103], although some reports pointed to reduced autophagy during the early response [104]. Interestingly, the up-regulation of pro-autophagic Atg5, Beclin-1, and LC-3 II could be blocked by the renin inhibitor aliskiren [102]. In the Ang II model, hypertrophic changes have also been associated with activated autophagocytosis in the heart, as suggested by an increased LC3 II/I ratio and up-regulated levels of Beclin-1 and Atg5 [105,106,107]. The activation of autophagy in the cardiac cells is believed to be a direct response to increased blood pressure in the heart. The role of mechanical stress in the induction of autophagy has been confirmed in CMs in vitro [99,100].

Activated autophagocytosis was recognized as playing an important role in disease progression. In the TAC model, on the one hand, the expression of *Becn1* was shown to control pro-hypertrophic and profibrotic responses in the heart, suggesting that autophagy enhances pressure-overload-induced heart failure [98]. On the other hand, mice with *Atg5* deficiency in CMs developed cardiac dysfunction and left ventricular dilatation one week after TAC, pointing to the cardioprotective role of autophagy in the hypertrophic heart [101]. Similarly, a study using cardiac-specific lysosomal deoxyribonuclease II deficient mice in the TAC model demonstrated that autophagy prevented myocarditis-mediated cardiac death by eliminating an excessive amount of mitochondrial DNA originating from damaged mitochondria due to hemodynamic stress [108]. In the case of the Ang II model, studies with *Atg5*^+/−^ mice also suggested that this autophagic response prevented exacerbated cardiac inflammation and fibrosis [106]. Furthermore, Ang II-induced hypertrophy is negatively regulated by the adipose tissue hormone adiponectin, which was shown to activate autophagy [109].

Experiments with the pharmacological activation of autophagy with rapamycin in the TAC and Ang II models confirmed the cardioprotective and antifibrotic roles of autophagocytosis in the development of hypertrophy [107,110]. In the TAC model, rapamycin improved certain remodeling parameters even in mice with established heart failure [111]. Furthermore, AMPK-dependent autophagy has been demonstrated to be a key mechanism behind the cardioprotective effect of the plant-derived compound corosolic acid in the TAC model [103]. It should be noted that some reports have suggested the suppression of autophagy after receiving cardioprotective treatments. In the TAC and Ang II models, for instance, treatment with melatonin, benzyl ester brozopine, or the calcium-sensing receptor inhibitor Calhex231 reduced ventricular hypertrophy, and this was associated with the inhibition of cardiac autophagy [112,113,114]. Moreover, in a transgenic mouse model of cardiac hypertrophy (heat shock protein 27 transgenic mice), the administration of the autophagy inhibitor wortmannin indicated the improvement of cardiac functions [115]. However, in the case of targeting non-autophagic pathways, the causative relationship must be confirmed.

Experimentally induced hypertrophic and fibrotic changes can spontaneously regress when disease-inducing agents are removed and ventricular blood pressure is stabilized. Published data suggest that autophagy plays an active role in the process of hypertrophy regression, as well. In the TAC model, treatment with rapamycin of mice with established hypertrophy could enhance the regression of hypertrophic changes and cardiac fibrosis, and improve heart function [116,117]. Data with conditional knockout of *Atg5* in CMs as well as *Becn1*^+/−^ mice confirmed the functional involvement of autophagy and pointed to the transcription factor FoxO1 as a key regulator of autophagy during the regression of cardiac hypertrophy [118,119]

### 8.3. Diabetic Cardiomyopathy

Diabetes represents another condition linking autophagy and cardiomyopathy. In humans, diabetes mellitus is recognized as an important risk factor for the development of heart failure. Some diabetic patients develop a diabetic cardiomyopathy phenotype characterized by interstitial fibrosis, CMs hypertrophy, and diastolic and systolic dysfunction. High glucose itself tends to inhibit autophagy, but it can also cause pro-autophagic mitochondrial dysfunction and oxidative stress. Therefore, the exposure of different cell types to high glucose may inhibit or activate autophagic flux depending on the context and cell type [120,121,122,123]. Importantly, an analysis of the hearts of type II diabetic patients showed elevated levels of the autophagy markers LC3 II and Beclin1 and an increased number of autophagosomes, indicating that, despite the high glucose levels in these patients, autophagy is activated by other mechanisms [124,125]. The ablation of insulin-producing pancreatic β cells by streptozotocin injection represents the most commonly used model of experimental diabetes. In this model, during the early phase (2–4 weeks), autophagy markers are elevated in the cardiac tissue, whereas during the late phase (8 weeks), characterized by cardiac fibrosis and systolic dysfunction, mTOR is up-regulated and autophagy is inhibited. *Beclin1*^+/−^ and *Atg16L1-HM* transgenic mice with reduced autophagic flux showed improved cardiac function as well as reduced interstitial fibrosis and CM apoptosis in the streptozotocin-induced diabetes model [126]. These data indicated the detrimental role of autophagy in diabetic cardiomyopathy. However, in contrast to the genetic model, pharmacological modulation of autophagy suggests a cardioprotective role. Accordingly, treatment with the autophagy inducers resveratrol, trehalose or metformin reduced cardiac fibrosis and improved cardiac function [121,127,128,129]. Metformin is a widely used anti-diabetic drug that lowers blood glucose levels by blocking gluconeogenesis; therefore, the cardioprotective effect in this model could, at least partially, be dependent on its glycoregulatory activity. Noteworthily, metformin showed similar anti-fibrotic and cardioprotective activity in normoglycemic δ-sarcoglycan deficient mice with established dilated cardiomyopathy [130]. In line with these data are the pharmacological inhibition of autophagy with chloroquine enhanced cardiac fibrosis, cardiomyopathy, and worsened heart function in streptozotocin-induced mice [131]. However, another study reported that chloroquine improved left ventricle diastolic function in this model [132].

Genetically obese db/db mice, which overeat because of a mutation in the leptin receptor gene, represent another animal model of diabetes. The 12-week old db/db mice develop increased cardiac fibrosis and moderate diastolic dysfunction and show low autophagy activity in the cardiac tissue [131]. The treatment of db/db mice with rapamycin or resveratrol increased autophagic activity and protected the heart from fibrotic changes and diastolic dysfunction, pointing to the cardioprotective role of autophagy in diabetic cardiomyopathy [131,133]. On the other hand, the autophagy inhibitor chloroquine worsened cardiac function, caused left ventricular dilatation, and increased cardiac fibrosis [131].

Of note, diabetic mice and rats are more susceptible to myocardial infarction and, in the ischemia-reperfusion model, develop greater infarct size [96,134]. The activation of autophagy either by the genetic overexpression of Atg5 and Lamp2 or pharmacologically with rapamycin or metformin reduced infarct size in diabetic animals [96,134]. Interestingly, the injection of rapamycin shortly before the reperfusion phase preserved postinfarction cardiac function and reduced fibrosis in diabetic rabbits subjected to ischemia-reperfusion [135].

### 8.4. Cardiotoxicity

Treatment with certain medications, particularly those used in the treatment of cancer, may lead to serious complications in the heart, eventually leading to heart failure. These cardiotoxic adverse effects can occur months or even years after the treatment. In fact, the risk of cardiotoxicity strongly limits the use of many potentially effective drugs. Doxorubicin is widely used in the clinic as a potent chemotherapeutic agent. However, its usage was reduced since it was shown that serious side effects such as heart failure might appear years after therapy [136]. In experimental animal models, doxorubicin triggers oxidative stress, causing CMs apoptosis and damage to the myocardium, which can lead to the development of cardiomyopathy and cardiac dysfunction [137]. Although autophagy markers are elevated in doxorubicin-treated animals, autophagic flux is blocked due to defective autophagolysosome formation [138,139,140,141]. This results in the lipocalin 2-dependent accumulation of autophagosomes, and mice deficient in lipocalin 2 are partly protected from the cardiotoxic effect of doxorubicin [142]. Furthermore, in the doxorubicin model, mice with defective autophagy, such as *Becn1*^+/−^ or following treatment with the autophagy inhibitor 3-MA, develop fewer autophagosomes and are protected from cardiomyopathy [138,140]. All these findings suggest that the doxorubicin-triggered accumulation of autophagosomes is detrimental. In this context, it might seem surprising that the activation of autophagy can be protective in the doxorubicin-induced model. Starvation prior to doxorubicin treatment partly restored the formation of functional autophagolysosomes and protected the mice from left ventricular dysfunction [141]. Similar cardioprotective effects were observed in mice or rats receiving pharmacological activators of autophagy, rapamycin, resveratrol, or metformin [49,139,143,144]. Furthermore, these data highlighted that the activation of autophagy inhibited apoptosis induced by doxorubicin. Thus, the activation of the physiological autophagic flux seems to represent a defense mechanism in response to cardiotoxic doxorubicin.

Isoproterenol-induced cardiomyopathy represents another example of a heart failure model. Isoproterenol is a nonselective beta-adrenergic agonist that was used in the clinic in the past to treat bradycardia and heart block. The treatment of mice with high doses of isoproterenol induces, however, myocardial necrosis, hypertrophy, fibrosis, and left ventricular dysfunction. These changes are associated with increased apoptosis and autophagic flux in the cardiac tissue [145,146,147,148]. In the isoproterenol model, a study reporting the cardioprotective effect of the CD47 antibody pointed to reduced apoptosis in the case of increased expression of autophagic markers [147]. This might suggest that the activation of autophagy induced by isoproterenol is beneficial for cell survival. On the other hand, the stimulation of autophagy with rapamycin worsened systolic function and enhanced cardiac fibrosis in isoproterenol-treated *Tlr4*^−/−^ mice [149]. However, there are not enough published data to clearly identify the effect of activated autophagy in the isoproterenol-induced heart failure model.

In summary, extensive research has been conducted to unravel the intricate role of autophagy in maintaining cardiac homeostasis and its involvement in heart failure. The findings from genetic models investigating autophagy-related genes are summarized in Table 1, while the impact of pharmacological treatments targeting autophagy is outlined in Table 2. Although the majority of studies support the cardioprotective role of autophagy, there are also conflicting findings suggesting alternative roles in certain contexts. These conflicting observations highlight the complexity of autophagy regulation in the heart and underscore the need for further investigation to fully elucidate its precise role in cardiac pathophysiology.

## 9. Autophagy in Cardiac Muscle Cells

**Figure 2:**
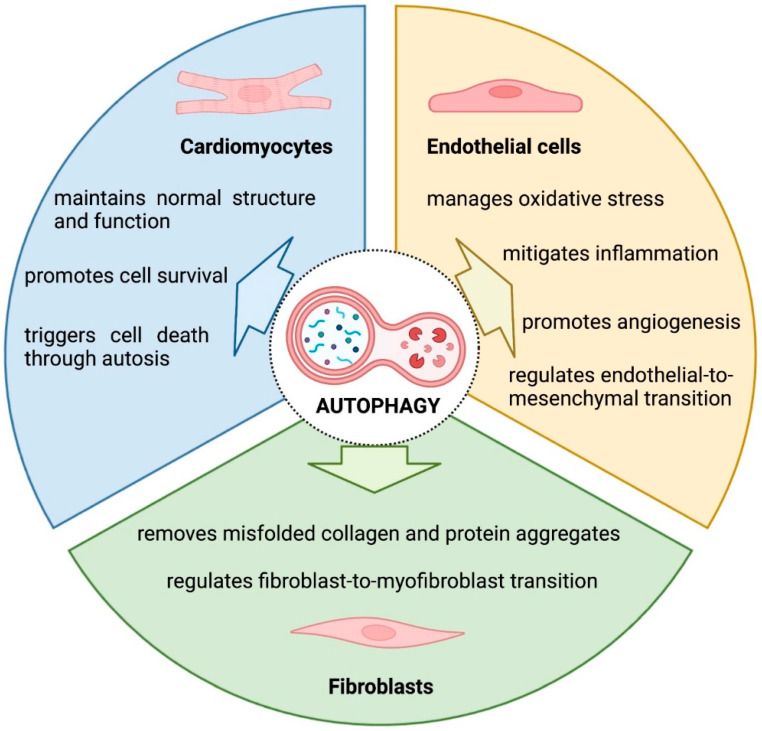
Summary of the impact of autophagy in main cardiac muscle cell types.

The heart is characterized by a high level of cellular diversity, which contributes to the organ’s structure and its ability to pump the blood. The main cardiac cell types are CMs, fibroblasts, and ECs, which represent >80% of all cells in the cardiac tissue [150,151,152]. The remaining cardiac cell types include smooth muscle cells, pericytes, macrophages, and neural cells. All these different cell types work in a coordinated manner to maintain a complex three-dimensional network and proper function of the heart. The main cellular processes regulated by autophagy in cardiomyocytes, cardiac fibroblasts, and ECs are highlighted in Figure 2.

### 9.1. Cardiomyocytes

CMs are the major components of mouse hearts responsible for their contraction. Under normal physiological conditions, CMs show constantly active, low-level autophagy. This process plays an important role in clearing damaged organelles and misfolded proteins. It enables the CMs to maintain normal structure and function [101,153]. CMs are considered one of the most sensitive cells in the body and have a limited capacity to regenerate and replace injured tissue; therefore, mechanisms protecting against their death play a particularly important role in this cell type. Cardiac stress conditions such as oxygen and nutrient deprivation, mechanical stress, or cytokine and growth factor signaling can activate autophagy in CMs [82,98,154,155,156,157]. This autophagy response can be protective or detrimental.

It is considered that stressed CMs activate autophagy as a protective mechanism to remove damaged organelles and protein aggregates to generate energy and maintain cell survival. This cardioprotective effect occurs at low and moderate stress levels and can prevent apoptosis and CMs loss. For example, in ischemic hearts high levels of autophagy markers in CMs correlate with low levels of myocyte apoptosis [154]. Such an anti-apoptotic effect of activated autophagic flux in CMs was also observed following exposure to cardiotoxic drugs [49,139,143,147]. Under hypoxic conditions, an autophagic response in CMs is also needed to maintain cardiac myofibrillar structure and prevent excessive fibrosis [62].

However, many types of stress induce not only autophagy but also CMs death, and therefore these two processes are interconnected. Death by autophagy and death with autophagy are two concepts that could be used to describe the relationship between CMs death and autophagy [158]. The first one indicates that autophagy causes cell death. One example of autophagic cell death, termed autosis, is characterized by initially excessive autophagic activity resulting in an accumulation of autophagosomes, autolysosomes, and vacuoles. Autosis has been observed in CMs in the ischemia-reperfusion model [84]. This form of cell death can be rescued by reducing autophagic flux [159]. Furthermore, when exposed to doxorubicin, autophagy has been implicated in CMs death, involving other mechanisms of programmed cell death such as apoptosis and necrosis [160]. The second point draws attention to the above-mentioned fact that dying CMs can activate autophagy for survival purposes. However, under conditions of extreme stress, these protective mechanisms may not be enough to prevent cell death. Summarizing, it seems that autophagy initially protects CMs from apoptosis, but the imbalance between the formation and degradation of autophagy components may lead to CMs death.

### 9.2. Cardiac Fibroblasts

Cardiac fibroblasts play a crucial role in the synthesis and deposition of the ECM, which provides structural support and mechanical stability to the heart [161]. Under physiological conditions, collagens, fibronectin, and other ECM proteins contribute to the organization and strength of the cardiac tissue. In the injured or failing heart, cardiac fibroblasts become activated, which often results in their uncontrolled proliferation and ECM overproduction. Cardiac fibroblasts are responsible for secreting collagens into the extracellular space. However, under certain circumstances, misfolded collagen proteins can accumulate within the cardiac fibroblasts, posing a potential toxic threat to cellular health. Studies focusing on mutations in the gene encoding collagen type I have shed light on the involvement of autophagy in the elimination of misfolded procollagen aggregates. It has been shown that autophagy, through its ability to engulf and degrade cellular components, including protein aggregates, plays a critical role in the clearance of misfolded procollagen and the maintenance of endoplasmic reticulum homeostasis [162]. In line with these findings, adrenergic stimulation, which is mediated by signaling molecules such as isoproterenol or noradrenaline, has been shown to enhance autophagy and collagen degradation in rat cardiac fibroblasts [163]. These data suggest that autophagy can facilitate the elimination of collagen or collagen-related aggregates in cardiac fibroblasts.

The activation of cardiac fibroblasts plays a crucial role in the pathophysiology of the injured or failing heart. This process is characterized by uncontrolled proliferation and excessive synthesis of the ECM proteins. In particular, the transformation of activated cardiac fibroblasts into the myofibroblast phenotype (defined by the increased expression of contractile protein alpha smooth muscle actin) represents a key step in the fibrotic remodeling of the heart. In vitro experiments showed that a classical TGF-b-dependent cardiac fibroblast-to-myofibroblast transition was associated with increased autophagic activity, and this process could be blocked by autophagy inhibitors [164,165,166,167]. Molecular insight into this process highlighted the important roles of PARP-1 and FOSL2 in the TGF-b-inducible model [166,167]. Of note, autophagy-dependent cardiac fibroblast-to-myofibroblast transition could also be triggered by the immunosuppressive agent cyclosporin A [168]. A study conducted in a mouse model has confirmed that enhanced autophagy in cardiac fibroblasts could regulate the fibrotic remodeling of the heart [167]. However, the precise mechanisms by which autophagy promotes fibrosis in cardiac fibroblasts are not yet fully understood. It is speculated that autophagy may contribute to the activation and persistence of myofibroblasts by regulating the turnover of intracellular components, promoting ECM deposition, and facilitating cellular remodeling processes.

### 9.3. Endothelial Cells

Cardiac ECs, which form the inner lining of blood vessels within the heart, have gained recognition for their critical role in maintaining vascular function and cardiac health. However, compared to the published research on autophagy in CMs and cardiac fibroblasts, the understanding of autophagy in cardiac ECs remains relatively limited. Nonetheless, emerging studies in non-cardiac ECs are shedding light on the potential significance of autophagy in endothelial function in heart failure.

The dysregulation of autophagy has emerged as an important factor contributing to endothelial dysfunction. Autophagy plays a crucial role in regulating ROS and NO productions in ECs. In general, autophagy acts as an antioxidative mechanism by removing damaged mitochondria in a process of mitophagy and other cellular components that generate excessive ROS [169]. The inhibition of autophagy disrupts the delicate balance between ROS production and clearance, leading to exacerbated oxidative stress and endothelial dysfunction [170]. Interestingly, it has been observed that ROS can induce autophagy in ECs as a protective response to counteract oxidative stress [171]. Autophagy also regulates the production of NO, a critical molecule involved in endothelium-dependent vasodilation and vascular homeostasis. Autophagy modulates the activity of endothelial NO synthase, the enzyme responsible for NO synthesis in ECs [172,173]. Accordingly, the inhibition of autophagy in ECs can diminish NO bioavailability and thereby compromise endothelial function [174]. All these findings highlight the key role of autophagy in maintaining endothelial homeostasis.

The regulation of ECs inflammation is another regulatory function of autophagy. Dysregulated autophagy can lead to the accumulation of damaged proteins and promote inflammatory signaling pathways, contributing to EC and vascular inflammation. For example, the inhibition of autophagy under shear stress conditions has been associated with an increased production of inflammatory cytokines [174]. Similar results have been observed in studies conducted under low-shear-stress conditions, where autophagy inhibition promotes the production of inflammatory cytokines, ECs apoptosis and senescence [175]. In line with these findings, the induction of autophagy in ECs has been found to exert an anti-inflammatory effect by downregulating inflammatory cytokines, adhesion molecules, and the NF-kB signaling pathway [176,177,178]. Collectively, these findings suggest that autophagy plays an important role in mitigating inflammation in ECs.

Autophagy also plays a significant role in the regulation of angiogenesis and neovascularization, essential processes involved in the formation of new blood vessels. Studies have demonstrated that the inhibition of autophagy, either through pharmacological intervention or genetic manipulation, effectively inhibits EC migration, angiogenesis, and pseudotube formation [179,180,181]. Furthermore, the overexpression of the essential autophagy-related protein or pro-autophagic pharmacological treatment have been shown to enhance angiogenic processes, reinforcing the pro-angiogenic role of autophagy in ECs [180,181]. This autophagy-induced angiogenesis was shown to be mechanistically mediated through the generation of ROS and the activation of the Akt signaling pathway [180,181,182].

Furthermore, autophagy has been associated with the endothelial-to-mesenchymal transition (EndMT), a process where ECs undergo a transformation into profibrotic mesenchymal cells. Studies have demonstrated that inhibiting autophagy, either through pharmacological means or via genetic manipulation, can induce EndMT in an interleukin-6-dependent manner [183]. Various biological, chemical, or mechanical stimuli can trigger the transition of ECs into a mesenchymal phenotype. For instance, in the case of the cardiotoxic drug doxorubicin, restoring autophagic flux has been found to mitigate EndMT [184] and suggests a protective role of autophagy in ECs. However, in the context of EndMT induced by advanced glycation end products, blocking autophagy was shown to alleviate this process [185].

In summary, although our understanding of autophagy in cardiac ECs is limited, the available data suggest that autophagy may serve as a significant regulator of EC function in the context of heart failure.

## 10. Future Directions

Autophagy has emerged as a key player in the context of heart failure, and extensive efforts have been dedicated to understanding its significance and intricate molecular mechanisms. To further investigate the role of autophagy in heart failure, several potential future directions can be pursued.

One important research direction is to enhance our understanding of the molecular regulation of autophagic processes. Integrated multi-omics approaches offer a promising platform to broaden our understanding of autophagy in heart failure. Techniques such as genomics, transcriptomics, proteomics, and metabolomics can be utilized to comprehensively analyze the regulatory networks associated with autophagy. By integrating data from multiple omics levels, we can gain insights into the molecular mechanisms underlying autophagy dysregulation and its impact on heart failure pathogenesis. Moreover, the application of advanced imaging techniques and noninvasive diagnostic tools can significantly contribute to better understanding of autophagy in heart failure. The visualization and quantification of autophagic flux would also allow the efficacy of autophagy-targeted interventions to be monitored.

Exploring pharmacological interventions that modulate autophagy in heart failure is another important avenue of interest. Testing autophagy inducers or inhibitors in preclinical models can allow the evaluation of their effectiveness in improving heart failure outcomes. Investigating the safety profile and potential side effects of these pharmacological interventions is a crucial aspect in drug development. Preclinical models can be used to evaluate the systemic effects of autophagy-modulating compounds, their interactions with other therapeutic agents commonly used in heart failure management, and their impact on other vital organs. By testing autophagy-modulating compounds in preclinical models and evaluating their safety, we are paving the way for the development of innovative therapies.

While the knowledge gained from preclinical models is indispensable, further research is needed to translate these findings into clinical applications. Future studies should therefore also focus on bridging the gap between preclinical research and heart failure patients in the clinic. Clinical studies involving patient cohorts and tissue samples would provide crucial insights into the specific alterations in autophagy pathways and their correlation with disease progression and patient outcomes. Furthermore, clinical trials with FDA-approved drugs with autophagy modulatory effects should be launched using carefully selected heart failure patient cohorts. It should be noted that autophagy inhibitors, such as chloroquine and hydroxychloroquine, have raised concerns regarding their impact on cardiac complications [186]. In particular, the use of hydroxychloroquine has been associated with an increased risk of major adverse cardiovascular events in patients with pre-existing heart failure [187]. Hence, the activation of autophagy might hold greater promise as a potential therapeutic approach. Currently, there are two ongoing phase I and phase II trials testing the use of metformin (trial no. NCT05093959) and rapamycin (Sirolimus, trial no. NCT04996719) in older adults with heart failure with preserved ejection fraction. The outcomes of these trials will provide valuable insights into the efficacy and safety of autophagy activation as a therapeutic strategy for heart failure patients.

Additionally, sodium-glucose cotransporter 2 (SGLT2) inhibitors, originally intended for glucose management in diabetic patients, have been shown to trigger autophagy ([188]). Notably, various clinical trials have demonstrated a reduced risk of cardiovascular mortality and hospitalization due to heart failure with the administration of SGLT2 inhibitors (Empagliflozin, Canagliflozin, and Dapagliflozin) ([189,190,191]). These findings further emphasize the rationale behind considering autophagy modulation as a potential therapeutic strategy for heart failure.

In summary, further research is essential to advance our knowledge in preclinical models and translate it into clinical applications. By pursuing these research avenues, we can better understand how autophagy contributes to heart failure and potentially uncover novel therapeutic approaches to improve the management of the disease and improve patient outcomes.

## Figures and Tables

**Table 1:** Summary of transgenic mouse models used to evaluate the role of autophagy in cardiac homeostasis and heart failure.

Disease Model	Transgen	Autophagy	Outcomes	Reference
**Homeostasis**				
None	*Lamp2* ^−/−^	↓	high premature mortality, surviving mice develop pathological changes in heart	[55]
None	*Lamp2* ^−/−^	↓	small vessels vasculopathy, accumulation of autophagic vacuoles	[56]
None	*Atg5* ^−/−^	↓	cardiomyopathy, systolic dysfunction	[57]
None	*Gsk3a^−/−^*	↓	cardiomyocyte loss, cardiomyopathy, cardiac dysfunction, shortened mouse lifespan	[58]
None	*Fbxo32^−/−^*	↓	accumulation of protein aggregates, cardiomyocyte apoptosis, age-related cardiomyopathy	[59]
None	*HSPB6^S10F^*	↓	age-related cardiomyocyte apoptosis, cardiac dysfunction, cardiac fibrosis, shortened mouse lifespan	[60]
None	Akt overexpression in cardiomyocytes	↓	age-related cardiac hypertrophy, interstitial fibrosis, contractile dysfunction	[61]
None	*MerCreMer^+^* x *Atg7^fl/fl^*	↓	contractile dysfunction, large vacuoles in the cross-section of cardiomyocytes, disorganized myofiber, cardiac fibrosis	[62]
None	*MerCreMer,* x *Atg5^fl/fl^*	↓	cardiomyopathy, cardiac dysfunction, shortened mouse lifespan	[63]
None	miR-199a overexpression in cardiomyocytes	↓	cardiomyopathy, cardiac dysfunction, cardiac fibrosis	[64]
None	*Ckm-Cre* x *Atg7^fl/fl^*	↓	cardiomyopathy, cardiac fibrosis	[71]
None	*Atg5* overexpression	↑	extended mouse lifespan	[65]
None	*Becn1* ^F121A/F121A^	↑	extended mouse lifespan	[66]
None	*FoxO3* overexpression	↑	reversible heart atrophy	[67]
**Heart Failure**				
AMI	*Becn1^+/−^*	↓	LV dilation, systolic dysfunction	[86]
AMI	*Becn1* ^+/−^	↓	more fibrotic scarring, worse cardiac function	[90]
AMI	*Becn1* ^+/−^	↓	reduced scar size, improved heart function	[91]
AMI	*IKKε^−/−^*	↑	increased infarct size, cardiomyocyte loss, worse cardiac function	[94]
I /R	*Atg7* ^−/−^	↓	aggravated cardiac injury, severe contractile dysfunction, increased cardiac fibrosis	[62]
I /R	Blockade of Hsp20 phosphorylation	↓	suppressed autophagic flux, increased infarct size	[78]
I /R	*Becn1* ^+/−^	↓	decreased size of myocardial infarction and area at risk	[82]
I /R	*Becn1* overexpression	↑	enhanced autosis, accumulation of autophagic vacuoles, exacerbated injury	[84]
Ang II	*Atg5^+/−^*	↓	increased cardiac fibrosis, enhanced cardiac inflammation	[106]
Ang II	*Apn^−/−^*	↓	increased cardiac fibrosis, no difference in cardiac function	[109]
Ang II	*MLC2vCre^+/−^* x *Atg5^fl/fl^*	↓	aggravated cardiomyopathy, increased cross-sectional area of cardiomyocytes, increased cardiac fibrosis	[118]
TAC	*MerCreMer^+^* x *Atg5^fl/fl^*	↓	LV dilatation, severe contractile dysfunction, increased cross-sectional area of cardiomyocytes	[101]
TAC	*MLC2vCre^+/−^* x *Atg5^fl/fl^*	↓	increased LV mass, decreased cardiac function, aggravated cardiomyopathy	[118]
TAC	*DDiT4L* overexpression	↑	mild systolic dysfunction, thinner ventricular walls, larger LV diastolic dimension	[68]
TAC	*Dnase2a^−/−^*	↑	LV dilatation, severe contractile dysfunction, increased cross-sectional area of cardiomyocytes, intermuscular and perivascular fibrosis, disorganized sarcomere structure	[108]
TAC	*FoxO1* overexpression	↑	reduced cardiac mass and cardiomyocyte cross-sectional area	[119]
DC (STZ)	*Beclin1^+/−^*	↓	improved cardiac function, reduced interstitial fibrosis, reduced CM apoptosis	[126]
DC (STZ)	*Atg16L1-HM*	↓	improved cardiac function, reduced interstitial fibrosis, reduced CM apoptosis	[126]
DC (STZ) I/R	*Atg5* overexpression	↑	decreased myocardial infarction, increased autophagic flux	[134]
DC (STZ) I/R	*Lamp2* overexpression	↑	decreased myocardial infarction, increased autophagic flux	[134]
DOX	*Beclin 1* ^+/−^	↓	maintained cardiomyocytes autophagic flux, preserved cardiac function, less pathological cardiac remodeling	[138]
DOX	*Mif* ^−/−^	↓	increased mortality, enhanced cardiomyocytes apoptosis, aggravated cardiomyopathy, decreased cardiac function	[139]
DOX	*Lcn2* ^−/−^	↑	reduced cardiomyopathy, improved cardiac function and decreased cross-sectional area of cardiomyocytes	[142]
ISO	*Tlr4* ^−/−^	↓	improved cardiac function, reduced cardiac fibrosis	[149]

Abbreviations: Ang II—angiotensin, AMI—acute myocardial infarction, DC—diabetic cardiomyopathy, I/R—ischemic/reperfusion IVSd—end-diastolic interventricular septal thickness, LV—left ventricular, STZ—streptozotocin, TAC—transverse aortic constriction, ↑—upregulation of autophagy, ↓—downregulation/suppression of autophagy.

**Table 2:** Summary of pharmacological modulators of autophagy tested in mouse models of heart failure.

Disease Model	Treatment	Autophagy	Outcomes	Reference
AMI	Bafilomycin A1 in GFP-LC3 mice	↓	increased infarct size, reduced myocardial ATP content, lower blood pressure	[72]
AMI	3-methyladenine in C57BL/6J mice	↓	increased infarct size, exacerbated cardiac fibrosis, unaffected cardiac function	[74]
AMI	Aspirin in C57BL/6J mice	↓	improved cardiac function, reduced cardiac fibrosis	[92]
AMI	Rapamycin in C57BL/6J mice	↑	reduced infarct size and cardiac fibrosis, improved cardiac function	[74]
AMI	Metformin in C57BL/6J mice	↑	improved hemodynamics, reduced inflammation	[76]
AMI	Trehalose in C57BL/6J mice	↑	improved systolic and diastolic function, reduced heart weight, reduced LV remodeling	[86]
I/R	Wortmannin in C57BL/6J mice	↓	increased myocardial infarct size	[85]
I/R	LncRNA CAIF in C57BL/6J mice	↓	attenuated myocardial infarct size, improved cardiac function, inhibited cardiomyocyte death	[93]
I/R	Rapamycin in *Hsp20*^S16A^ mice	↑	rescued post-ischemic function	[78]
I/R	Metformin in C57BL/6 mice	↑	reduced infarct size, improved cardiac function, reduced mortality	[81]
Ang II	Chloroquine in C57BL/6J mice	↓	aggravated cardiomyopathy, worsened cardiac function, exacerbated cardiac fibrosis	[107]
Ang II	Rapamycin in C57BL/6J mice	↑	attenuated cardiac fibrosis, reduced cardiomyopathy, reversed cardiac dysfunction	[107]
TAC	3-methyladenine in C57BL/6J mice	↓	reduced cardiomyopathy, improved cardiac function, decreased cross-sectional area of cardiomyocytes	[102]
TAC	Aliskiren in C57BL/6J mice	↓	reduced cardiomyopathy, improved cardiac function, decreased cross-sectional area of cardiomyocytes, inhibited ECM changes	[102]
TAC	Brozopine in C57BL/6J mice	↓	reduced cardiomyopathy, improved cardiac function, decreased cross-sectional area of cardiomyocytes, reduced cardiac fibrosis	[113]
TAC	Melatonin in C57BL/6 mice	↓	reduced cardiomyopathy, decreased cross-sectional area of cardiomyocytes	[114]
TAC	Corosolic acid in C57BL/6J mice	↑	reduced cardiomyopathy, improved cardiac function, decreased cross-sectional area of cardiomyocytes, reduced cardiac fibrosis	[103]
TAC	Rapamycin in C57BL/6 mice	↑	improved cardiac function	[111]
TAC	Rapamycin in FVB/N mice	↑	reduced cardiomyopathy, decreased cross-sectional area of cardiomyocytes, decreased LV diameters, unchanged cardiac function	[110]
TAC	Rapamycin in FVB/N mice	↑	decreased HW-BW ratio, LW-BW ratio, myocyte cell size, LV end-systolic dimensions, improved cardiac function	[116]
TAC	Rapamycin in FVB/N mice	↑	decreased LV mass index, LV wall thickness, HW, cardiomyocyte size, collagen deposition	[117]
ISO	Aspirin in Balb/c mice	↓	improved cardiac function, reduced cardiac fibrosis	[92]
ISO	Rapamycin in *Tlr4*^−/−^ mice	↑	increased cardiac dysfunction, exacerbated cardiac fibrosis, increased myocyte loss	[149]
Cardiomyopathy	Wortmannin in *Hsp27* overexpressing mice	↓	improved cardiac function, no changes in heart size	[115]
DC (db/db)	Chloroquine in *lepr*^db^/*lepr*^db^ mice	↓	worsened cardiac function, increased cardiac fibrosis	[131]
DC (db/db)	Chloroquine in *lepr*^db^/*lepr*^db^ mice	↓	improved LV diastolic function, decreased cardiac fibrosis reduced cardiomyocyte apoptosis	[132]
DC (STZ)	Metformin in C57BL/6J mice	↑	improved cardiac function, reduced cardiac fibrosis	[121]
DC (STZ)	Resveratrol in CD1 mice	↑	improved cardiac function, reduced cardiac fibrosis, reduced mortality	[127]
DC (STZ)	Trehalose in C57BL/6J mice	↑	improved cardiac function, reduced cardiac fibrosis, decreased myocardial enzymes, (LDH, AST, CK, CK-MB), decreased cell apoptosis	[129]
DC (STZ)	Metformin in *Sgcd*^−/−^ mice	↑	reduced cardiomyopathy, improved cardiac function, attenuated LV hypertrophy, myocardial fibrosis and cardiomyocyte hypertrophy, increased autophagic flux	[130]
DC (db/db)	Rapamycin in *BKS-Leprdb* mice	↑	decreased myocyte size, reduced fibrosis	[133]
DC (STZ) I/R	Rapamycin in C57BL/6 mice	↑	decreased myocardial infarction, increased autophagic flux	[134]

Abbreviations: Ang II—angiotensin, AMI—acute myocardial infarction, DC—diabetic cardiomyopathy, DOX—doxorubicin, ECM—extracellular matrix, HW-BW—heart-to-body weight ratio, LncRNA CAIF—long noncoding RNAs cardiac autophagy inhibitory factor, LW-BW—lung-to-body weight ratio, I/R—ischemic/reperfusion, ISO—isoproterenol, LV—left ventricular, STZ—streptozotocin, TAC—transverse aortic constriction, ↑—upregulation of autophagy, ↓—downregulation/suppression of autophagy.

## Data Availability

Not applicable.
